# White-Nose Syndrome Confirmed in Italy: A Preliminary Assessment of Its Occurrence in Bat Species

**DOI:** 10.3390/jof7030192

**Published:** 2021-03-09

**Authors:** Laura Garzoli, Elena Bozzetta, Katia Varello, Andrea Cappelleri, Elena Patriarca, Paolo Debernardi, Marco Riccucci, Angela Boggero, Carolina Girometta, Anna Maria Picco

**Affiliations:** 1 Department of Earth and Environmental Sciences, University of Pavia, 27100 Pavia, Italy; carolinaelena.girometta@unipv.it (C.G.); annamaria.picco@unipv.it (A.M.P.); 2 S.Te.P. Stazione Teriologica Piemontese, 10022 Carmagnola, Italy; teriologi@gmail.com (E.P.); info@centroregionalechirotteri.org (P.D.); 3 CNR-Water Research Institute (IRSA), 28922 Verbania, Italy; angela.boggero@cnr.it; 4 Department of Specialised Diagnostic, Istituto Zooprofilattico Sperimentale del Piemonte, Liguria e Valle d’Aosta, 10154 Turin, Italy; elena.bozzetta@izsto.it (E.B.); Katia.Varello@izsto.it (K.V.); 5 Department of Veterinary Medicine, University of Milan, 26900 Lodi, Italy; andrea.cappelleri@unimi.it; 6 Mouse and Animal Pathology Laboratory (MAPLab), Fondazione UniMi, 20139 Milan, Italy; 7 Zoological Section «La Specola», Museum of Natural History of the University of Florence, 50125 Florence, Italy; marco.riccucci@gmail.com

**Keywords:** Chiroptera, *Pseudogymnoascus destructans*, *Geomyces destructans*, white-nose disease, hibernacula, maternal roost, *Myotis*

## Abstract

Although no mass mortality has been recorded so far, the precise demographic effect of white-nose syndrome (WNS) on European bats still remains to be ascertained. Following the first isolation of *P. destructans* in Italy, further surveys were performed to assess the distribution of the fungus in NW Italy and its effects on bats. Data were collected from March 2019 to April 2020 at sites used for hibernation (six sites) and/or for reproduction (four sites) in Piedmont and Aosta Valley. A total of 138 bats, belonging to 10 species, were examined to identify clinical features possibly related to the fungal presence. Culture from swabs and the molecular identification of isolates confirmed the presence of *P. destructans* in bats from five sites, including two maternal roosts. Dermal fungal infiltration, the criterion to assess the presence of WNS, was observed in biopsies of bats belonging to *Myotis blythii*, *M. daubentonii*, *M. emarginatus* and *M. myotis*. This is the first report of the disease in Italy. The results suggest a greater susceptibility to the infection of the genus *Myotis* and particularly of *M. emarginatus*, possibly due to the long length of its hibernation period. Other fungal dermatophytes were also observed.

## 1. Introduction

An emerging fungal disease of bats—white-nose disease (WND) or white-nose syndrome (WNS)—has devastating effects on North American bat populations. Millions of bats have died so far of the infection; in some species, declines have exceeded 95%, and entire populations have been extirpated [1]. Indeed, the disease may represent the largest mammalian wildlife mortality event in recorded history [2]. It is caused by the psychrophilic fungus *Pseudogymnoascus destructans* (formerly *Geomyces destructans*), a pathogen firstly discovered in Upstate New York in 2006 [3,4]. The fungus infects hibernating bats while their immunological response is limited by the torpor [5]. It initially grows on the skin’s surface and then progresses by invading the underlying tissues, where hyphae can form cup-like epidermal erosions and invade hair follicles, sebaceous glands, and the nearby connective tissue [5,6]. Lesions might be particularly severe on the wing and tail membrane, whose tissues are involved in gas exchanges, fluid balance, thermoregulation, and immune functions [7,8,9]. This provokes a cascade of physiological consequences, among which are an increased metabolic rate, dehydration, and electrolyte imbalances, resulting in frequent arousals, the premature depletion of fat reserves, starvation, and eventually death [10,11,12,13,14,15]. The diagnosis of the disease requires both the demonstration of the presence of the fungus in the skin—via PCR or through culture—and histopathology to identify the presence of the typical dermal lesions [16].

Genomic studies indicate that *P. destructans* is native to Eurasia [17,18]. This is also consistent with photographic evidence [19,20,21,22] and its isolation from museum specimens: it has recently been retrieved from a *Myotis bechsteinii* collected on 9 May 1918, in the Forêt de Russy, Centre-Val de Loire, France [23]. The fungus is thought to have been introduced to North America in the first years of the present century [3,17,20]. Although the route of introduction is still unknown, human-mediated transport seems to be the likely cause, as it has also been suggested for the arrival of the fungus in Washington state in 2016 [24,25]. *Pseudogymnoascus destructans* is thought to expand only clonally in North America [18,26,27,28], while in Europe it has the potential for sexual recombination [29].

Globally, *P. destructans* has been found on at least 62 bat species; in Eurasia, it has been detected on 41 host-species of bats and, in more than 76% of them, the diagnostic lesions of WNS were also documented [1].

Although no mass mortality has been reported [20], the precise effects of the disease on European bats still remain to be ascertained. Pikula and coauthors [6] demonstrated that the disease can also be fatal to European bats. Moreover, deaths related to the disease may remain unrecognized, in particular if bats die after their emergence from the hibernacula.

In 2019, some of the authors of the present paper reported the first record of *P. destructans* in Italy [30]. The fungus was retrieved through the culture of swabs collected from some *Myotis emarginatus* captured in a cave in Piedmont (NW Italy) and then molecularly confirmed. None of the bats showed visible fungal growth, and, in the absence of histological analyses, the occurrence of WNS was not ascertained.

As bats in Europe and Italy are facing many threats, understanding the role of pathogens is important to plan strategies for their conservation. To our best knowledge, there are no other studies about the presence of *P. destructans* in Italy. This work aims to contribute to the knowledge on the pathogen and its effects on bats in NW Italy.

## 2. Materials and Methods

### 2.1. Sample Collection

**Figure 1:**
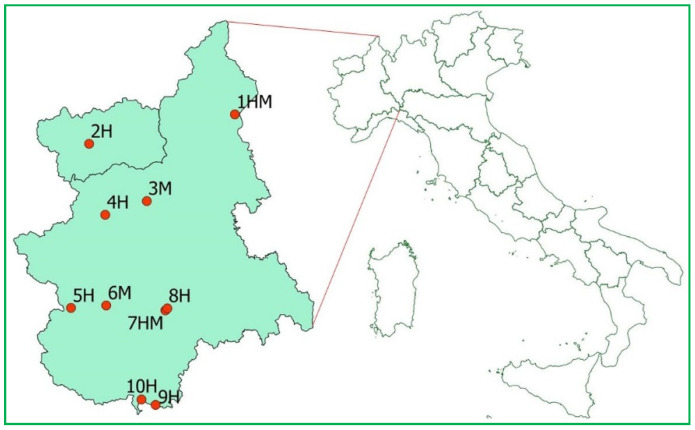
Sites sampled for the occurrence of *P. destructans* in Piedmont and Aosta Valley (NW Italy). H—hibernation site; M—maternity roost.

Data were collected from March 2019 to April 2020 at sites used for hibernation (H, 6 sites) and/or as maternity roosts (HM and M, 4 sites) in Piedmont and Aosta Valley (NW Italy, Figure 1). Captures of bats and samplings were carried out under license by trained personnel (see the “Institutional Review Board Statement” section for further details). The cave in which the occurrence of *P. destructans* had already been ascertained (site 5H, Figure 1) was included and monitored through repeated surveys in order to verify whether other bat species of the site, together with *M. emarginatus*, hosted the fungus. The site is used for hibernation by multiple bat species and the fact that only *M. emarginatus* was found positive for the fungus during the previous surveys might be due to the small sample of individuals examined and the late period of the surveys (June and July) [30,31]. The other sites were visited 1–2 times, in periods when the emergence from hibernation or the first arrivals to the maternity sites were expected to occur (based on previous knowledge).

In a few cases (8 individuals), sampling was limited to the collection of swabs for fungal isolation from torpid bats at the end of the hibernation period, without handling them.

In the other cases (130 individuals), bats were mistnetted at the entrances of the hibernacula or captured inside maternity sites. For each bat, we recorded features possibly related to fungal presence, such as visible fungal growths or signs of damage on ears (irregular edges) and wings (holes, irregular pigmentation and necrotic edges, detected by inspecting the patagium under direct light and by transillumination). Body mass and forearm length were recorded and later used to compare the conditions of the captured individuals by means of a Body Condition Index (BCI (g/mm) = 100× body mass/forearm length) [32]. Body mass values were also compared with values considered “normal” in the literature, referring to Lanza [33] and Dietz and Kiefer [34].

Sterile cotton swabs (packed in shockproof round bottom sterile polypropylene tubes) were used to take a superficial skin sample as in Garzoli et al. [30]. Swabs for fungal isolation and wing biopsies were collected from a subsample representative of all the species investigated. We did not conduct biopsies on individuals that showed physical damage due to traumatic events and on those with large holes in their wings to avoid affecting their survival. We did not collect swab samples during the sampling in June at site 5H because the data collected during previous surveys were sufficient to confirm the presence of the fungus in the investigated bat species. Wings were examined using long-wave UV light, as shown by Turner et al. [35]; we used 10-watt 370–375 nm (Irtronix, Inc., 20900 Normandie Ave., Torrance, CA, USA) and 15-watt 380–400 nm (Onforu, Mengzhituo Technology Co., LTD. Shenzhen, China) LED sources, but they both elicited little or no fluorescence. For this reason, biopsies (one for each sampled bat) were mainly taken from depigmented areas. We used 4 mm sterile punches for larger species (forearm > 50 mm) and 3 mm punches for the other species.

### 2.2. Fungal Isolation and Identification

Swabs were preserved at 4 °C during transport and then immediately processed in the laboratory. Samples were processed according to Gargas and co-authors [4], as described in Garzoli et al. [30]. Briefly, under laminar flow, swabs were gently streaked onto the surface of 9 cm Petri dishes containing Sabouraud Dextrose Agar medium (SAB, Biolife, Milan, Italy). Plates were incubated in the dark at 4 °C and checked weekly for up to two months. Each fungal morphotype was isolated in pure culture and maintained at 4 °C. Strains in pure culture (on SAB) are preserved at the fungal collection of the Laboratory of Mycology and Plant Pathology, Department of Earth and Environmental Sciences, University of Pavia and are available on request. The growth of the colony at 4 °C, the shape and dimension of conidia and conidiophores were considered diagnostic characters for strains ascribable to *P. destructans*. Findings were confirmed through molecular analyses by sequencing the nuclear ribosomal nrDNA partial regions (ITS) using the universal fungal barcode primers ITS1/ITS4 [36,37]. Newly generated sequences were compared to those available in public databases (GenBank -nblast, Mycobank) and deposited at NCBI (Accession number: from MW447503 to MW447508). Identifications were then confirmed by evaluating morphological consistency with molecular findings.

### 2.3. Histopathological Analyses

Samples for histology were immediately fixed in 10% neutral buffered formalin. In the laboratory, they were dehydrated in a graded series of ethanol and then embedded in paraffin. Samples were cut in 4 ± 2 µm serial sections and stained with haematoxylin–eosin (HE) standard stain to characterize the inflammatory infiltration and with Periodic Acid Schiff stain (PAS) to identify the fungal hyphae. The slides were observed microscopically at increasing magnification (10×, 20×, 40×) by a light microscope (Zeiss Axio Scope A1, Frankfurt am Main, Germany).

## 3. Results

A total of 138 bats, belonging to 10 species, were examined. None of them showed visible fungal growth. The body mass of the captured individuals (*n* = 130) did not differ from the values reported in the literature as normal [33,34], with the exception of four *R. ferrumequinum*, three of which were slightly lighter (16.5–17.5 g) and one, an adult female, which was clearly underweight (12.9 g) and showed the lowest value of the BCI index recorded for the species (21.83) (Table 1 and Table S1).

The majority of bats (99%, 129 bats) showed some visible signs of wing damage (Table S1), but these were generally mild, with depigmentation covering, at worst, 30% of the patagium and no notable loss of membrane area.

Swabs and biopsies were collected from 94 and 72 individuals, respectively.

Cultural analyses of swabs confirmed the presence of *P. destructans* in 23 bats from 5 sites (Table 1 and Table S1), 3 used as hibernacula (2H, 4H, 5H) and 2 maternal roosts (3M, 6M). The positive species were *M. blythii* (75.0% positive, *n* = 4 individuals sampled), *M. daubentonii* (14.3%, *n* = 7), *M. emarginatus* (78.6%, *n* = 14) and *M. myotis* (47.1%, *n* = 17).

Histopathological features possibly referable to the fungus were observed in biopsies from 11 bats, captured at 3 of the same sites (2H, 5H, 3M) and 2 of the other sites (1HM, 7HM) (Table 1 and Table S1).

**Figure 2:**
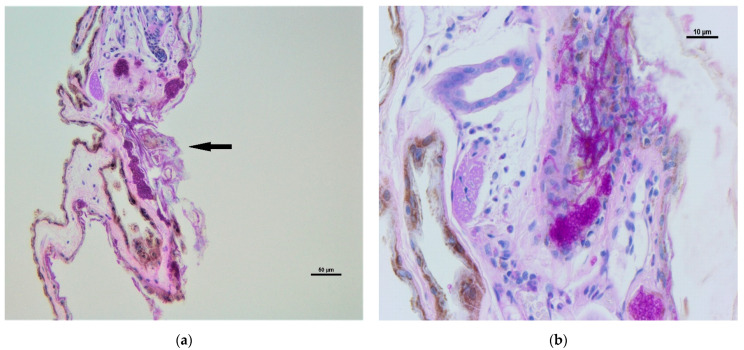
Wing membrane: (**a**) epidermal erosion and ulceration with moderate dermal infiltration (arrow) and fungal hyphae; (**b**): detail of fungal hyphae infiltration in glands and derma (arrows) with mild inflammatory infiltration (mononucleated cell and rare neutrophils). Periodic Acid Schiff (PAS) stain.

The dermal infiltration of fungal hyphae was observed in five biopsies, obtained from individuals of *Myotis blythii* (1), *M. daubentonii* (2), *M. emarginatus* (1) and *M. myotis* (1); swabs confirmed the presence of *P. destructans* in all the individuals, with the exception of one *M. daubentonii*. The infiltration appeared mild to moderate with epidermal erosions and ulcers, fungal hyphae in hair follicles and glands (Figure 2A) with mild to moderate inflammatory infiltration represented by neutrophils and mononucleated cells (Figure 2B).

The other six PAS+ biopsies showed only fungal skin-surface colonization. They were taken from bats belonging to *M. emarginatus* (3), *M. myotis* (2) and *M. capaccinii* (1). The latter was the only individual of this species that showed histopathological features possibly referable to the fungus, but it was not possible to verify the result through mycological analyses because the culture of the swab failed due to contaminants.

Overall, both isolation and histopathological evaluation retrieved the fungus on five bats belonging to *Myotis blythii*, *M. daubentonii*, *M. emarginatus*, and *M. myotis*; three of them also showed signs of inflammation, while two did not. In four cases, fungal hyphae were revealed by PAS staining, but the fungi detected through swab culture did not include *P. destructans*, belonging instead to *Alternaria* sp., *Aureobasidium pullulans* var. *melanigenum*, *Cladosporium* sp., *Fusarium* sp. and *Penicillium* sp. In another 11 individuals, swabs demonstrated the presence of *P. destructans*, but the pathogen was not detected at histopathology; in two of these bats (one *M. daubentonii* and one *M. emarginatus* from site 5H), the allied species *P. pannorum* was also detected. Colonies of this species grew faster than *P. destructans*, reaching a colony diameter of 40 mm in 15 days even at 4 °C. Microscopical features were consistent with those reported in the literature [38]. In particular, conidia were obovoid, slightly echinulate, mostly 3 × 2.5 µm, and lacking the distinctive curved-shaped we observed in the pathogenic species (see also Garzoli et al. [30]).

The other bats examined (belonging to *Rhinolophus ferrumequinum*, *R. hipposideros*, *Barbastella barbastellus*, *Hypsugo savii* and *Miniopterus schreibersii*) did not resulted positive either to mycological or histopathological analyses, even when sampled at the same sites and sampling sessions in which positive species were detected. Several other fungal taxa were detected, mainly comprising members of genera *Penicillium*, *Cladosporium*, and *Beauveria*. Sterile mycelia were also recorded and isolated in pure culture.

As for bats belonging to species positive for the disease, the frequency of external signs of damage possibly related to *P. destructans* did not differ between positive and negative individuals. Furthermore, signs of damage also characterized individuals belonging to species in which the fungus was not recorded.

The only female individual of *R. ferrumequinum*, which was clearly underweight according to BCI, resulted negative to either swab and histopathological analyses. The lower BCI value was therefore likely related to causes other than WNS.

## 4. Discussion

This is the first study that confirms, in addition to the presence of its causative agent, the actual occurrence of white-nose syndrome in Italy. Dermal infiltration, i.e., the criterion to confirm the disease [16], was observed in two sites and four species, *M. blythii*, *M. daubentonii*, *M. emarginatus*, and *M. myotis*, already reported in the literature to be affected by the disease in Europe [39].

Puechmaille and co-authors [20] suggested a low prevalence of the disease in the Mediterranean region due to the short hibernation period and the high temperatures recorded in hibernacula, and Martinkova and co-authors [40] presented models predicting that the occurrence and invasiveness of *P. destructans* infection in Italy are marginal. Nonetheless, even if these statements proved to be true, surveying the Italian situation should be considered important since observing the effects of *P. destructans* where it is expected to be less invasive may help to identify the species more sensitive to the pathology, and to understand how the infection works.

We demonstrated that *P. destructans* is widespread in North-Western Italy, even at low altitudes, being present in at least 5 out of the 10 surveyed sites. Moreover, we detected the fungus not only at hibernacula, but also at two maternal roosts, where we retrieved the pathogen on female individuals of *M. myotis* in the early spring. The occurrence of *P. destructans* at nursery sites has been reported by several authors [41,42,43,44]. The fungus is considered to be psychrophilic, and its prevalence decreases during summer because of the high body temperature of active bats [42]. However, it has recently been proven that it is able to survive in vitro at 24 °C for 60 days, and even at 37 °C for 15 days [45]. Huebschman and co-authors [44] have demonstrated that it can be transmitted to juveniles inside nurseries. Consequently, we highlight the need for monitoring maternal colonies, with priority for those that use colder roosts, in order to evaluate the persistence of the fungus and its effects.

We detected fungal lesions through histopathologic analysis in a minority of the biopsies examined, but it should be remarked that, due to the almost complete lack of fluorescence, the biopsies were conducted without UV-guidance. The fluorescence of WNS lesions is a consequence of the production and bioaccumulation of the secondary metabolite riboflavin (vitamin B_2_), which also plays a role in the progression of skin necrosis; it is observed only after invasive growth and in the presence of cup-like erosions [46]. The absence of fluorescence that we observed might be due to different causes: a low fungal load and a consequent scarce production of the metabolite, the occurrence in bat skin of a microbiome capable of limiting the fungus growth and metabolism, or a shorter exposure time of the bat tissues to dangerous fungal loads. Regarding the latter, it should be noted that the winters proceeding the samplings were particularly mild, and this might have reduced the negative effects of the fungus by shortening the hibernation period or allowing foraging during arousals.

We detected inflammatory processes in only some of the infected bats. The lack of inflammation is generally acknowledged as common in WNS onset [5], even though inflammatory processes can dramatically increase during the post-hibernation period as demonstrated by Meteyer and co-authors [47], who suggested that affected bats may undergo a form of Immune Reconstitution Inflammatory Syndrome (IRIS).

Histopathological examination revealed fungal presence on the skin surface of one *M. capaccinii* (not reported as a host species of *P. destructans* in the literature), but we did not identify the fungus because the swab culture failed.

Neither swab culture nor histopathology detected signs of *P. destructans* colonization in bats belonging to *R. ferrumequinum*, *R. hipposideros*, *H. savii*, *B. barbastellus* and *M. schreibersii*, although some of them (*H. savii*, *B. barbastellus*, *M. schreibersii* and some of the *R. ferrumequinum*) were from sites where the fungus was ascertained to occur. *Rhinolophus hipposideros*, *B. barbastellus* and *M. schreibersii* are reported as host species of the fungus in the literature [7,48], and we must highlight that our results are not conclusive, as we have examined a small sample of individuals. Nevertheless, our data suggest a greater susceptibility to the disease of the genus *Myotis* compared to the other bats examined.

Within the genus *Myotis*, we observed a high percentage of individuals positive for *P. destructans* in *M. emarginatus*, possibly because of the long hibernation period of this species [30,34].

We recorded the presence on studied bats of other fungal species whose pathogenic potential should be further investigated. Among them, *Pseudogymnoascus pannorum* has already been reported as an opportunistic pathogen causing skin damage in humans [49]. Nevertheless, we did not observe inflammation or external damages in one of the two positive bats, and in the other, which showed irregular wing pigmentation, *P. destructans* was also detected. Another fungal taxon known for its opportunistic behavior is *Scopulariopsis brumptii* [50], found on four individuals investigated. Nonetheless, histopathology did not evidence significant wing lesions related to its presence. The finding of members of genera *Cladosporium* and *Penicillium* is not surprising, as these are airborne, saprophytic fungi commonly retrieved in environmental samples or in hibernacula [51]. These genera were also retrieved on bat carcasses from Italy [52]. Worthy of interest is the finding of *Beauveria brongniartii* in 8% of samples, even if the fungus does not seem to be related to histopathological damage. Further investigation is needed to clarify the role of this and other fungi and to determine the role of secondary fungal infections in the survival of bats once they arrive at summer roosts.

Following the detection of *P. destructans* at site 5H [30], we set up a decontamination protocol for the visitors of the site (http://www.parcomonviso.eu/media/2486371a.pdf (accessed date 8 March 2021)). Even if the primary cause of fungal dispersal at a local level is likely due to bats [1], human-mediated dispersion should not be overlooked. Visitors of infected sites can carry viable fungal spores [53] and transport them over long distances, reaching previously uncontaminated areas [24,54] or even spreading haplotypes with higher virulence among already infected sites.

Further work is needed in order to understand the diffusion of *P. destructans* in Italy and its effects on bats, but, in any case, the national authorities should take action to prevent the human-mediated dispersal of this and other fungal pathogens, particularly through more effective cross-border controls.

## Figures and Tables

**Table 1:** Bats examined and individuals investigated through culture (positive/negative for *P. destructans*) and histological (positive/negative for fungal presence) analyses. In brackets, the number of individuals for which a dermal infiltration by hyphae was observed.

Species	Site and Role	N. Examined	Mean BM ± SD	Mean BCI ± SD	N. Swabs		N. Biopsies	
			(g)	(g/mm)	−	+	−	+
*Rhinolophus ferrumequinum*	2H	6	19.6 ± 1.96	33.8 ± 3.00	6	0	5	0
	8H	4	18.7 ± 2.08	32.7 ± 3.42	4	0	4	0
	9H	2	15.2 ± 3.18	25.8 ± 5.56	2	0	2	0
*Rhinolophus hipposideros*	9H	4	4.8 ± 0.34	12.4 ± 0.70	4	0	4	0
	10H	5	Nr	Nr	5	0	0	0
*Myotis blythii*	4H	1	Nr	Nr	0	1	0	0
	5H	4	22.3 ± 0.77	38.6 ± 0.78	1	2	3	1 (1)
*Myotis capaccinii*	1HM	13	9.2 ± 0.61	22.0 ± 1.27	8 *	0 *	9	1
*Myotis daubentonii*	5H	10	6.7 ± 0.55	17.6 ± 1.31	6	1	3	2 (2)
*Myotis emarginatus*	4H	3	7.6 ± 1.04	19.0 ± 2.15	0	3	3	0
	5H	49	7.3 ± 0.97	18.1 ± 1.88	3	8	6	4 (1)
*Myotis myotis*	2H	1	24.5	40.1	0	1	0	1
	4H	2	26.6 ± 1.06	42.3 ± 0.35	0	2	2	0
	5H	1	23	38.1	1	0	1	0
	7HM	2	23.9 ± 2.97	40.2 ± 4.04	2	0	1	1
	3M	4	24.5 ± 0.88	39.5 ± 1.59	0	4	2	1 (1)
	6M	7	26.9 ± 1.95	42.4 ± 2.53	6	1	7	0
*Barbastella barbastellus*	2H	5	7.8 ± 0.47	19.9 ± 0.89	5	0	3	0
	5H	13	8.5 ± 0.74	21.8 ± 1.53	11	0	6	0
*Hypsugo savii*	2H	1	Nr	Nr	1	0	0	0
*Miniopterus schreibersii*	5H	1	12.5	27.5	1	0	0	0

Abbreviations and symbols: H—Hibernation sites; M—Maternity roosts; * The culture of swabs from a further 5 individuals sampled failed due to contaminants; BM—body mass; BCI—Body Condition Index; SD—Standard Deviation; Nr—not recorded (data were collected by swabbing torpid bats, without handling them).

## Data Availability

The data presented in this study are available in the present article and in Supplementary Table S1.

## Supplementary Materials

The following are available online at https://www.mdpi.com/2309-608X/7/3/192/s1, Table S1: Presence of *Pseudogymnoascus destructans* and other fungal taxa in bats of NW Italy. Histopathological and cultural analyses results are reported.
